# Can We Prepare IVF Culture Media Two Days Before Ovum Pick up Without Affecting Embryological Parameters? A Retrospective Case-Matched Study

**Published:** 2019

**Authors:** Alessio Paffoni, Elisabetta Ballabio, Sabrina Cesana, Stefania Ferrari, Hilda Wyssling, Marco Claudio Bianchi

**Affiliations:** 1- ASST Lariana, Infertility Unit, Cantù, Italy; 2- Fondazione IRCCS Ca’ Granda Ospedale Maggiore Policlinico, Milan, Italy

**Keywords:** Culture medium, Embryo, Embryology, IVF

## Abstract

**Background:**

According to several laboratory protocols and specific conditions, *in vitro* fertilization (IVF) dishes with culture media can be prepared 24 *hr* in advance compared to routine protocols. However, it is not clear if this procedure can affect embryological outcomes.

**Methods:**

A nested case-control study was done in a cohort of couples undergoing IVF at the Infertility Unit of the ASST Lariana from August 2016 to July 2018. Cases were patients undergoing ovum pick up after a laboratory day off. Controls were patients undergoing ovum pick up after working days from Monday to Thursday. Culture media for oocyte culture and insemination were prepared about 42 and 18 *hr* before oocyte retrieval for cases and controls, respectively. Cases and controls were matched with a 1:2 ratio (for age, inseminated oocytes, length of stimulation). The “Good-Quality-Index” (GQI) was the main outcome to be compared between the two groups and was defined as good quality transferred or cryopreserved embryos on day 2 or 3+number of good quality blastocysts/inseminated oocytes.

**Results:**

A total of 76 cases and 152 matched controls were enrolled. The median GQI was equal to 33.0% (IQR: 20.0–50.0%) and 33.0% (IQR: 25.0–50.0%), in cases and controls, respectively (p=0.40). Study groups and GQI were not significantly correlated (correlation coefficient r=0.047, p=0.48). Main embryological parameters and cumulative pregnancy rates were similar between the two groups.

**Conclusion:**

Our data support the vision that culture media can be prepared 24 *hr* in advance compared to routine protocols without affecting embryological outcomes.

## Introduction

The need to perform ovum pick up for *in vitro* fertilization (IVF) on weekends has been debated for a long time. Since the early days of IVF, several strategies have been proposed to avoid clinical and laboratory staff to be on duty seven days a week (1). With the use of gonadotropin releasing hormone (GnRH) agonist protocols, certain flexibility in planning the ovum pick up procedure has been obtained, and in many clinics, oocyte retrievals can be performed only on weekdays without affecting the outcomes (2–6). On the contrary, the use of GnRH antagonist protocols may represent a matter of concern since gonadotropin administration is dependent on naturally occurring menses. Delaying ovum pick up for more than one day in antagonist cycles has been reported to result in unfavorable outcomes (7, 8). However, advancement or delay in triggering ovulation for just one day is considered to be neutral with regard to IVF outcomes (9). Of note, a randomized controlled trial failed to demonstrate a negative effect of postponing hCG administration up to two days (10). Since the embryo transfer can be easily scheduled between day 2 and day 5 without evident effects on IVF outcomes (11, 12), the clinical activities can be suspended in some settings during weekends, according to the number of cycles performed; the higher the number of cycles, the lower the chance to avoid ovum pick up and/or embryo transfer on weekends.

Despite the efforts made to achieve precise cycle scheduling, the laboratory staff has to follow different rules in order to avoid the need to be on duty on weekends. In fact, according to the IVF schedule for the entire week and even in the absence of oocyte retrievals or embryo transfers, embryologists are supposed to be on duty in order to check fertilization, to perform embryo biopsy or cryopreservation based on the developmental stage of cultured embryos, to make culture media renewal or to prepare culture media for the next day. The use of single culture media with uninterrupted protocols and time lapse monitoring of embryos can offer a more effective time management for embryologists since their use can limit the need for the laboratory staff to be at work at precise fixed times (13–15). However, a day off in the lab is quite rare since a series of concurrent situations need to be verified, such as no embryos to be moved to new culture media or to be evaluated for biopsy or cryopreservation and no ovum retrieval the day before (need for checking fertilization) or the day after (need for preparing culture media). The latter point deserves further consideration in this context. To ensure proper temperature and pH of culture media and overlaid mineral oil, as stated by most manufacturers, culture dishes are to be pre-equilibrated not less than 4–6 *hr* at 37*°C* in CO_2_ incubator. Since ovum pick up is generally performed in the morning, embryologists prepare dishes for oocyte culture and insemination on the day before oocyte collection (Day1) (16). In our IVF unit, standard operating procedures allow lab staff to prepare those dishes 24 *hr* in advance in case of no other lab activities the day before ovum pick up. However, the concern has not been clearly addressed that this procedure can potentially influence IVF results, and this study aims to provide more information on this topic. It has been reported that a variety of factors, such as volume of media, temperature and time used in preparation could have an impact on the quality of commercially available culture media (17). Of note, ammonium can accumulate in ready-to-use embryo culture media during storage at 2–8*°C* and particularly during incubation at 37*°C* degrees in a time-dependent manner. The deterioration of culture media during storage and incubation suggests that their capacity to support embryo development can decrease over time (18).

The aim of the present study was to investigate if the preparation of IVF media 24 *hr* in advance (before a day off in the lab, thus two days before oocyte retrieval) compared to controls (the day before oocyte retrieval) can be potentially harmful for gametes or embryos.

## Methods

### Study design:

This is a nested case-control study in a cohort of couples undergoing IVF at the Infertility Unit of the ASST Lariana from August 2016 to July 2018 aimed to evaluate if oocyte retrievals performed after a laboratory day off (cases) have reduced embryo developmental potential compared to those performed after working days from Monday to Friday (controls). Exclusion criteria were as follows: 1) women aged 43 years or older at the time of oocyte retrieval; 2) having less than two oocytes suitable for IVF. Cases and controls were matched with a 1:2 ratio for age (±2 years), range of inseminated oocytes (2–3; 4–6, 7–15 oocytes) and length of stimulation (±0 days). The case-control matching procedure was performed using the MedCalc Statistical Software (version 18.6; MedCalc Software bvba, Belgium).

All patients involved in the study provided prospective written consent for use of their anonymized medical records for scientific/quality assurance purposes such as this study. The work has been carried out in accordance with the Code of Ethics of the World Medical Association (Declaration of Helsinki) and Uniform Requirements for manuscripts submitted to biomedical journals. The ethical approval was obtained (Comitato Etico ASST Sette Laghi).

### Clinical protocol:

All women selected for IVF in our unit underwent a clinical assessment and a baseline transvaginal ultrasound during the menstrual cycle preceding the controlled ovarian hyperstimulation (COH). Patients were managed according to standardized clinical protocols for ovarian hyperstimulation with recombinant FSH (rFSH) alone or rFSH combined with recombinant LH (rLH) or highly purified human menopausal gonadotrophin alone (HP-hMG) in combination with daily administration of GnRH agonist (Decapeptyl, Fertipeptyl) or antagonist (Cetrotide, Orgalutran) according to basal characteristics and biomarkers of ovarian reserve. The dosage of gonadotrophins (150 to 350 *UI/die*) was determined by the woman’s age, prior response to ovarian stimulation and ovarian reserve status assessed by antral follicle count and serum Anti-Mullerian Hormone (AMH) levels. In case of excessive, insufficient or abnormal follicular growth, the cycle could be cancelled before oocyte retrieval. Ultra-sound scans (US) and estradiol measurements were performed to assess follicular and endome-trial development. Triggering was induced in the presence of two or more follicles ≥17 *mm* in diameter, with the majority of follicles being ≥14 *mm*. Oocyte retrieval was performed 34 *hr* after triggering ovulation and embryo transfer was performed two to three days after oocyte insemination. One or two embryos were replaced per cycle. Embryo transfer was postponed through embryo vitrification in the presence of any risks of ovarian hyperstimulation syndrome (OHSS) and one or two embryos destined for embryo transfer were cryo-preserved on day 3 of culture. Non-replaced embryos were cultured up to day 7 after insemination in order to cryopreserve supernumerary viable ones at the blastocyst stage. Women with vitrified embryos were scheduled for elective single embryo transfer in a subsequent cycle. Frozen embryo transfers were performed in a natural cycle, 2–5 days after ovulation (detected with the use of daily US), according to the developmental stage of the embryo at the time of cryopreservation. No luteal phase support was given. Alternatively, vitrified embryos were transferred using hormone replacement therapy, mainly if women had irregular menstrual cycles or if the monitoring of the natural cycle failed. Serum hCG assessment to detect pregnancy was performed 12 (±2) days after embryo transfer. Women with positive hCG values underwent transvaginal sonography two and three weeks later. Clinical pregnancy per cycle was defined as the presence of at least one intrauterine gestational sac obtained with the fresh or subsequent cryopreserved embryo transfer deriving from the same oocyte aspiration procedure.

### Laboratory protocol:

The day before oocyte retrieval, a 4-well dish (IVF dish) containing 500 microliters per well of ready-to-use medium (Sequential Fert, Origio, Målov Denmark or Continuous Single Culture medium, Irvine Scientific, Santa Ana, USA) with human serum albumin over-laid with mineral oil was prepared for every patient. This IVF dish was used to rinse and culture oocytes for 4±1 *hr* after collection and for insemination with 35.000 motile spermatozoa per well and subsequent overnight culture in case of standard IVF. Moreover, a dish containing 25 microliter droplets of culture medium (Quinn’s Advantage Cleavage Medium, Origio or Continuous Single Culture medium, Irvine Scientific) was prepared to culture ICSI-treated oocytes immediately after injection (C1 dish). For oocyte retrieval procedures performed after a day off in the laboratory (study group), the above described dishes (IVF and C1 dishes) were prepared 24 *hr* in advance compared to ordinary conditions, *i.e*. about 42 *hr* before oocyte retrieval. The control group is made of patients undergoing oocyte retrieval from Monday to Friday with IVF dishes of culture media prepared in the afternoon of the day before oocyte retrieval (about 18 *hr*). The two groups did not differ in other aspects of laboratory procedure and in particular in the preparation of dishes used for subsequent steps of culture.

The presence of pronuclei was checked 18–20 *hr* after insemination and zygotes were transferred to a new dish with 40 microliters drops of culture media prepared in the afternoon of the oocyte retrieval (C2 dish). Fresh embryo transfer was performed on day 3 or less frequently, for reasons of convenience, on day 2 after oocyte retrieval. One or two embryos were replaced according to female age and embryo quality. Embryos with the highest quality score were chosen for embryo transfer. In case of postponed embryo-transfer, embryos destined for replacement were cryo-preserved on day 3 after oocyte retrieval. Super-numerary embryos were cultured to the blasto-cysts stage in 40 microliter droplets of fresh medium (Quinn’s Advantage Blastocyst, Origio, or Continuous Single Culture medium, Irvine Scientific) up to day 7. A group culture strategy was used with up to four embryos in the same droplet from the pronuclei stage to the blastocyst stage. The vitrification protocol with Cryotop (Kitazato, Japan) according to Kuwayama (19) was used to cryopreserve embryos.

Embryo quality was recorded on the day of embryo transfer and, in case of supernumerary embryos, on day 5, 6 and 7. The Alpha/ESHRE consensus (20) was used to classify embryos at the cleavage and blastocyst stage. A “good quality embryo” on day 2 or 3 was defined in the presence of an appropriate cell number, fragmentation less than 10% and stage-specific cell size with no evidence of multinucleation. Good quality blasto-cysts were defined in case of expansion corresponding to grade 2 or more with both inner cell mass and trophectoderm classified as type 1 or 2.

The “Good Quality Index” (GQI) was the main outcome and was defined as follows: (number of good quality transferred or cryopreserved embryos on day 2 or 3+number of good quality blasto-cysts)/number of inseminated oocytes.

### Statistical analysis:

Analysis of data was performed using the Statistical Package for Social Sciences (SPSS 18.0, Chicago, Illinois, USA). Statistical significance was set at p<0.05. Data were compared using unpaired Student’s t-test, chi-squared test, Fisher’s exact test or Mann-Whitney test. Exact Fisher’s confidence intervals were computed for rates and reported as 95% Confidence Interval (95% CI). Normally distributed variables are presented using means±standard deviations, whereas skewed data are presented using medians and interquartile ranges (IQR) between square brackets. A point-biserial correlation was run between cases/controls and GQI. Linear and logistic regression were run to predict continuous or dichotomous dependent variables from independent variables. Odds ratios (OR) are reported with their 95% CI.

To obtain the sample size of the study, for power calculation purposes based on our previous data, 20% of relative decrease of the GQI in cases compared to controls (from 35% to 28%, respectively) was considered clinically important. The sample size was then calculated considering type-I and type-II errors equal to 5% and 20%, respectively. About 500 and 1000 inseminated oocytes were needed in cases and controls, respectively. A 24-month period was deemed sufficient to achieve this sample size.

## Results

In the study period, 652 cycles were identified. The application of exclusion criteria was as follows: 67 (10%) cycles were excluded for maternal age and 93 (14%) cycles were excluded for insufficient number of inseminated oocytes. Among the remaining 492 cycles, 79 (16%) cases were identified. For three cases, matching controls were not available; therefore, results are shown for 76 cases with 152 matched controls. Oocyte retrievals of cases were performed on Mondays (n=74, 97%) or Friday (n=2, 3%) after a non-working day; for controls, they were performed after working days on Mondays (n=24, 16%), Tuesday (n= 30, 20%), Wednesday (n=41, 27%), Thursday (n= 25, 16%) or Friday (n=32, 21%).

Baseline characteristics of the enrolled patients did not significantly differ between the two groups (Table 1). As shown in table 2, the IVF cycles had similar characteristics and outcomes between cases and controls. Among non-pregnant women, 3 out of 47 (6%) cases and 11 out of 99 (11%) controls had available frozen embryos to be transferred in the future (p=0.55).

**Table 1.** Baseline characteristics of the selected couples

**Characteristics**	**Cases n=76**	**Controls n=152**	**p**
**Age (years)**	36.3±3.7	36.4±3.8	0.88
**BMI (*Kg/m*^2^)**	23.1±4.7	23.2±4.6	0.88
**Duration of infertility (years)**	4.3±2.0	4.4±2.3	0.75
**Patients with previous pregnancies**			0.61
with miscarriage	17 (22%)	31 (20%)	
with delivery	3 (4%)	11 (7%)	
**Number of previous IVF cycles**			0.65
0	45 (59%)	81 (53%)	
1–2	25 (33%)	59 (39%)	
3–5	6 (7%)	12 (8%)	
**FSH (*mIU/ml*)**	7.7±3.0	7.1±2.2	0.08
**AMH (*ng/ml*)**	2.1 (1.3–3.1)	1.9 (1.0–3.6)	0.73
**Sperm concentration (10^6^/*ml*)**	47 (6–83)	32 (8–80)	0.14
**Primary indication for IVF**			0.62
Female	15 (20%)	20 (13%)	
Male	16 (21%)	35 (23%)	
Female – Male	14 (18%)	32 (21%)	
Unexplained	31 (41%)	65 (43%)	

Data is reported as Mean±SD, Median (IQR) or Number (%).BMI: Body Mass Index. IVF: In Vitro Fertilization. FSH: Follicle Stimulating Hormone. AMH: Anti-Mullerian Hormone

**Table 2.** Characteristics of the IVF cycles in cases and in controls

**Characteristics**	**Cases**	**Controls**	**p**

	**N= 76**	**N=152**	
**Protocol of ovarian stimulation**			0.89
Protocol with GnRH agonists	11 (14%)	21 (14%)	
Protocol with GnRH antagonists	65 (86%)	131 (86%)	
**Total amount of FSH used (IU)**	1983±867	2067±946	0.52
**Duration of stimulation (days)**	9.1±1.8	9.1±1.8	1.00
**Number of follicles ≥ 14 *mm***	7.4±3.3	6.9±3.8	0.33
**Oocyte retrieved**			0.69
1–4	17 (22%)	42 (28%)	
5–9	39 (51%)	73 (48%)	
>9	20 (26%)	37 (24%)	
**Insemination**			0.65
Standard IVF	47 (62%)	99 (65%)	
ICSI	25 (33%)	42 (28%)	
Both	4 (5%)	11 (7%)	
**Number of inseminated oocytes**	5 (4–8)	5 (4–7)	0.57
**Fertilization rate (%)**	74 (53–100)	67 (50–83)	0.40
**Culture medium**			0.39
Sequential	59 (78%)	110 (72%)	
Single	17 (22%)	42 (28%)	
**Cancelled or delayed embryo-transfer^a^**	12 (16%)	33 (22%)	0.38
No viable embryos	1 (1%)	5 (3%)	0.67
Freeze-all	11 (14%)	28 (18%)	0.58
**No of transferred embryos per patient^a^**			0.56
1	24 (37%)	43 (36%)	
2	40 (63%)	76 (64%)	
**No of good-quality transferred embryos per patient^a^**			0.16
0	4 (6%)	19 (16%)	
1	33 (52%)	52 (44%)	
2	27 (42%)	48 (40%)	
**Pregnancies^a^**			
Serum hCG positive	22 (29%)	40 (26%)	0.67
Clinical pregnancy	21 (28%)	35 (23%)	0.45
**No of cryopreserved embryos**			0.10
0	50 (66%)	78 (51%)	
1–2	20 (26%)	53 (25%)	
3–5	6 (8%)	21 (14%)	
**No of cycles with subsequent frozen embryo transfer**	19 (25%)	51 (34%)	0.22
**Cumulative clinical pregnancies per cycle**	29 (38%)	53 (35%)	0.63
Singletons	26 (90%)	46 (87%)	1.00
Twins	3 (10%)	7 (13%)	
**Pregnancies ongoing (≥13 weeks) or to term**	20 (69%)	43 (79%)	0.28
**Overall implantation rate**	33/126 (26%)^b^	60/247 (24%)	0.71

a In the fresh cycle.

b In one cycle a pregnancy was obtained both in the fresh (abortion) and in the frozen embryo transfer. Data is reported as mean±SD or number (%) or median (IQR). IVF: In Vitro Fertilization; hCG: Human Chorionic Gonadotropin

Using standard IVF or ICSI, 441 and 853 oocytes were inseminated in cases and controls, respectively. On the day of embryo transfer, 103/441 (23%, 95%CI: 20–28%) and 195/853 (23%, 95%CI: 20–26%) good quality embryos were recorded, respectively (p=0.84). Moreover, a total of 38 and 99 good quality blastocysts were cryo-preserved giving a GQI equal to 32.0% (95%CI: 27.8–36.5%) and 34.5% (95% CI: 31.3–37.8%) in cases and controls, respectively (p=0.37). The median GQI was 33.0% (20.0–50.0%) and 33.0% (25.0–50.0%), respectively (p=0.40).

Study groups and GQI were not significantly correlated with point-biserial correlation coefficient r=0.047, p=0.48. A linear regression analysis confirmed that the association between GQI and the status of case/control was not statistically significant (B=0.024, p=0.48).

A multiple regression was run to predict GQI from age (female and male), AMH, number of inseminated oocytes, duration of stimulation, sperm concentration, and type of culture medium. The multiple regression model weakly, but statistically significantly, predicted the GQI F (8, 219)= 2.245, p=0.025, adj. R^2^=0.042). The regression coefficient associated with the status of case/control was slightly higher (from 0.024 to 0.028) after adjusting for above mentioned variables. Among considered variables, the number of inseminated oocytes added statistical significance to the prediction, p<0.05. Regression coefficients and standard errors can be found in table 3.

**Table 3.** Summary of multiple regression analysis to predict the “Good Quality Index”

**Variable**	**B**	**SEB**	**β**	**p**
**Intercept**	0.672	0.203		
**Case (value=1) – Control (value=2)**	0.028	0.034	0.054	0.41
**Age (female)**	−0.010	0.005	−0.151	0.07
**Age (male)**	0.002	0.003	0.056	0.49
**No of inseminated oocytes**	−0.023	0.006	−0.244	0.001
**Days of stimulation**	0.001	0.009	0.005	0.94
**Sperm concentration (10^6^/ml)**	0.001	0.000	0.126	0.06
**Culture medium (1=sequential; 2= single)**	−0.011	0.037	−0.019	0.80

B= Unstandardized Regression Coefficient; SEB=Standard Error of the Coefficient; β= Standardized Coefficient

The crude OR for cumulative pregnancy in cases compared to controls was 1.153 (95%CI: 0.651–2.039). After adjusting for confounding factors (mentioned above), the OR was 1.077 (95%CI: 0.585–1.982).

## Discussion

From the results of our study, it appears that when the unique scheduled activity in the IVF laboratory is preparation of culture dishes for the day after, the lab staff can have a day off, by means of preparing media the day before, achieving similar embryological outcomes. Our results are quite novel since this specific aspect has been given scanty consideration in the literature and can be considered reassuring for those who have the opportunity to have a day off for the entire IVF lab staff. It is likely that this opportunity is related to the number of cycles performed in the IVF center since the higher the number of cycles, the lower the chance to have a day without medical or embryological activities. Of note, in our center (performing about 500 fresh and frozen cycles per year), at least 50% of Sundays can be considered as “non-working” according to the scheduled activities. Since at least 50–60% of IVF clinics in Europe perform a similar or lower number of cycles per year (21), it is believed that our data can be useful to a large proportion of people involved in IVF activities.

The issue of programming IVF cycles in order to avoid weekends has been addressed since the early days of IVF (1), mainly focusing on ovulation trigger and timing of embryo transfer, but in general, the needs of the laboratory staff have received far less attention than they deserve.

It has been reported that embryo culture media can deteriorate during storage (22, 23), and since they have a key role in embryo development providing a proper environment with possible long-term consequences, it is of utmost importance to accumulate data regarding quality and safety. However, available data from the mentioned studies are derived from analyses on long-term storage or considering time from production date to usage (18). Here, in the absence of proof to the contrary, a difference in pre-incubation time as short as 24 *hr* does not have significant effects on the quality of culture media. Of note, culture media used for zygotes up to day 3 are generally used for 48 *hr* of culture plus pre-incubation time; a condition similar to that of IVF dishes used for our cases in the present study.

The design of the study gave two groups with comparable basal characteristics, and our results can be considered reasonably unbiased. In fact, the matching criteria were chosen in order to limit the effect of possible confounding factors on the main outcome. In addition to age, which has been reported to influence embryo quality (24, 25), length of stimulation and number of available oocytes were used in order to control for individual response to ovarian stimulation and for possible modifications in the timing of triggering in order to avoid oocyte retrievals during weekends. Moreover, the multiple regression analysis also allowed for the determination of the overall fit of the model and the relative contribution of each of the predictors to the total variance explained. In this case, additional variables were included for their possible contribution to embryonic development, namely age of the male partner and sperm concentration (26–28) and type of culture medium (29).

Of note, as suggested by the regression analysis, the GQI was not significantly affected by basal characteristics but the number of available oocytes.

The main clinical outcomes such as clinical pregnancy rate per cycle and cumulative implantation rate, were similar between groups. Although this data seems to be reassuring, confirming our main findings, it should be noted that the present study was not designed to highlight differences in pregnancy rates and that the power of the study was inadequate to draw conclusions on this specific aspect.

Some shortcomings of our study have to be recognized. First, this is a retrospective study, and although it was performed in a matched case-control setting with comparable basal characteristics, it can not be concluded that untested variables could influence the results. As usual, in these study protocols, unbalanced presence of confounding factors may explain the differences, therefore results should be considered with precautions.

Second, the study was designed with the main outcome of good quality embryos per inseminated oocytes. This is a widely used strategy for IVF cycle evaluation, but it has to be mentioned that it suffers from subjectivity and when studying the effect of a procedure on clinical outcome, the preferable endpoint should be childbirth. Moreover, since age of culture media has been reported to be associated with birthweight of IVF babies (18, 29), neonatal outcomes should be taken into account as well. Of note, these endpoints require a very large sample size since differences between cases and controls are expected to be small. Third, the predicted sample size has not been entirely achieved. In fact, the actual number of considered oocytes was 10–15% lower than expected. It has to be recognized that this aspect did not dramatically lower the power of the study (from 80% to 73%) and that the inclusion of further cycles in the past was not feasible due to significant changes in standard operating procedures. On the contrary, during the study period, no changes were made to laboratory procedures or staff. Finally, two main culture strategies (single and sequential) were used with ready-to-use formulation, and no differences were seen when considering this variable. However, caution is needed when extrapolating our results to laboratories using different culture conditions or type of media. In fact, it is conceivable that variables such as type of storage and incubation, composition of media, protein supplementation and type of plastics could play a role.

## Conclusion

In conclusion, the key message of our study is that, given the above mentioned limitations, culture media can be prepared two days before oocyte retrieval without a relevant effect on embryological parameters. Many embryologists could take advantage of these data when planning activities in order to avoid the laboratory staff to be on duty seven days a week. Further data are warranted in order to clarify if effects smaller than those supposed in the present study can exist and to shed more light on safety aspects of similar procedures in terms of the health of newborns.
